# Assessing the representativeness of large medical data using population stability index

**DOI:** 10.1186/s12874-025-02474-9

**Published:** 2025-02-21

**Authors:** Sheng-Chieh Lu, Wenye Song, Andre Pfob, Chris Gibbons

**Affiliations:** 1 Department of Symptom Research, The University of Texas MD Anderson Cancer Center, 6565 MD Anderson Blvd, Houston, TX 77030 USA; 2 Department of Obstetrics & Gynecology, Heidelberg University Hospital, Im Neuenheimer Feld 672, Heidelberg, 69120 Germany; 3 National Center for Tumor Diseases (NCT)and, German Cancer Research Center (DKFZ), Im Neuenheimer Feld 280, Heidelberg, 69120 Germany

**Keywords:** Population stability index, Sample representativeness, Big data analytic

## Abstract

**Background:**

Understanding sample representativeness is key to interpreting findings from epidemiological research and applying these findings to broader populations. Though techniques for assessing sample representativeness are available, they rely on access to raw data detailing the population of interest which are often not readily available and may not be suitable for comparing large datasets. In reality, population-based data are often only available in an aggregated format. In this study, we aimed to examine the capability of population stability index (PSI), a popular metric to assess data drift for artificial intelligence studies, in detecting sample differences using population-based data.

**Method:**

We obtained United States cancer statistics from the National Cancer Institute's Surveillance, Epidemiology, and End Results (SEER) database. We queried the SEER 17-registry research database to obtain cancer count data by age, sex, and cancer site groups from the rate sessions of the SEER*State incidence database for 2000 and 2015 – 2020. We then calculated PSI scores to estimate yearly data distribution shift from 2015 to 2020 for each variable. We compared the PSI results to the Chi-Square and Cramér's V tests for the same comparisons.

**Results:**

Scores for PSI comparing age, sex, and cancer site distribution between years ranged widely from 2.96 to less than 0.01. In line with our expectations, we found moderate to substantial differences in cancer population characteristics between 2000 and all other included years using PSI. Despite small effect sizes (Cramér's V 0.01 – 0.09), Chi-Square tests were significant for most comparisons, indicating likely type-I error caused by our large sample.

**Conclusions:**

Population stability index can be used to examine sample differences in healthcare studies where only binned data are available or where large datasets may reduce the reliability of other metrics. Inclusion of PSI in epidemiological research will give greater confidence that results are representative of the general population.

**Supplementary Information:**

The online version contains supplementary material available at 10.1186/s12874-025-02474-9.

## Background

In descriptive epidemiological studies, the representativeness of study samples is a cornerstone of generalizability and the application of findings to wider populations [1, 2]. It is well-established in the literature that poor sample representativeness leads to biased associations and/or suboptimal policy decision-making [3, 4]. Although researchers suggested that sample representativeness is not essential for causal relationship studies, sample representativeness assessment is necessary to ensure the proper application of the inference [5].

As data volume and the availability of population-based, real-world data increase, classic approaches for sample representativeness evaluation suffer from the over-powering issue of catching subtle, clinically meaningless differences between samples [6]. Epidemiologists have developed representativeness assessment metrics that provide better estimation of differences in large samples [7–10]. An example is Representativity indicators (R-indicators) that was first developed to estimate the differences between responders and non-responders in survey studies [8] and later applied to other studies for sample representativeness assessment [6]. R-indicators estimate overall sample representativeness based on the standard deviation of sample propensities [7]. However, the use of raw data of R-indicators can be a challenge when comparing study samples to population-based data for sample representative assessment as most population-based data, such as the National Cancer Institute's Surveillance, Epidemiology, and End Results (SEER) data, are only available in a post-aggregated format without additional access permission.

Despite the use of different terminology, the importance of sample representativeness is also highlighted by artificial intelligence (AI) and machine learning (ML) studies leveraging high-dimensional, huge-volume data [11, 12]. AI and ML fields use the term “data drift” or “concept drift” to describe the existence of differences in variable distributions between the sample used to train a model and the sample fed to the model for prediction. Therefore, concept drift and lack of sample representativeness are conceptually the same. As model performance can significantly decrease when feature drift happens [12], most, if not all, ML solutions highlight the need to detect drift after model deployment and offer various ways for automatic concept drift detection [13].

Population index stability (PSI) is a sample distribution distance-based statistic for measuring sample similarity [14, 15]. PSI measures the distribution differences in each class of a variable between samples and provides an overall score of the variable by summing the scores of each class. As such, PSI accepts only categorical variables, and numeric variables need to be binned to enable the use of PSI. The possible score of PSI ranges from 0 to 1, with a larger value representing greater differences in the variable between samples. A general rule adopted in practice to interpret a PSI result is: PSI < 0.1 represents no difference, PSI > = 0.25 indicates a significant difference, and any score between the two represents a slight difference [14].

Population index stability is a widely used metric in AI and ML fields to determine whether a predictive model needs refinement due to data changes over time [14]. However, the discussion on using PSI in healthcare research is limited. It is unclear whether PSI can be an alternative to established representativeness metrics when raw reference sample data is unavailable. The purpose of this study was to examine the capability of PSI in detecting differences in population-based samples. Specifically, we applied PSI to assess distribution changes in age, gender, and cancer types of the U.S. cancer population over time using SEER data.

## Methods

For this study, we extracted sex, age, and cancer type data of the U.S. cancer population from the Surveillance, Epidemiology, and End Results (SEER) [16]. We calculated PSI for each variable to compare the populations between all possible year-pairs across 2015 and 2020. We also extracted and compared data from the year 2000 to all other years to evaluate whether PSI could capture differences in data distributions that we hypothesized were likely to have occurred in a 15–20 year timeframe. We examined the PSI results by comparing them to the results from Chi-Square tests. This research was deemed exempt from ethical review because of the use of publicly-available anonymous data without human subject involvement.

### Data

We obtained U.S. cancer population statistics from the SEER database for this study. The SEER database, supported and maintained by the National Cancer Institute, collects comprehensive, population-based U.S. cancer incidence and survival data alongside cancer patient demographics, tumor information, diagnosis, and treatment data since 1973. The database is updated yearly and used to support oncology research and inform policy decision-making throughout the USA [17]. We downloaded aggregated data using the SEER*Stat Software (version 8.4.3).

We queried the SEER 17-registry research database submitted in November 2022. We obtained cancer count data by age, sex, and cancer site groups from the rate sessions of the SEER*State incidence database. For cancer site groups, we extracted incidence rate and patient count data for Lung, Breast, Colorectal, Genitourinary, and Melanoma. We obtained data for 2000 and 2015—2020.

### Population Stability Index (PSI)

We calculated PSI for sex, age group, and cancer type using the equation $PSI={\sum }_{i=1}^{k}\left({O}_{i}-{E}_{i}\right)\times \text{ln}\left(\frac{{O}_{i}}{{E}_{i}}\right)$, where k represents the total number of categories for the variable of interest, O is the percentage of patients in a category in the scoring sample, and E is the percentage of patients in a category in the reference sample [15]. We used the equation to calculate the PSI scores to estimate yearly data distribution shifting from 2015 to 2020 for each variable. For instance, we used age-group data from 2015 as a referencing sample and from 2016 as a scoring sample to calculate the PSI for the age-group data distribution change estimate. As no definitive gold standard reference for sample representativeness exists, we used PSI to compare the distribution differences in selected variables between years with the early year in each comparison as a reference sample to depict relative sample similarity for each year-pair.

We manually computed PSIs to ensure the compatibility of the calculation with aggregated data we obtained from the SEER database. We used widely used cut-off points of 0.1 and 0.25 in informatics literature to interpret PSI results, with PSI < 0.1 representing no distribution differences between samples, PSI > = 0.1 and < 0.25 meaning moderate differences, and PSI > = 0.25 indicating significant differences [18, 19].

We tested PSI in a scenario where the differences in age, sex, and cancer group composition of the U.S. cancer population between years are expected and well-studied to ensure that there were sample differences for the PSI to detect. However, we expected that data distribution changes in consecutive years would be less notable and would not raise concerns about the presentation of ignorable sample differences. Therefore, we expected that PSI would not detect action-required data changes in any consecutive year comparison, but Chi-square tests may still flag the differences due to the large power. To demonstrate the capability of PSI in detecting data changes, we compared data from each year to data from 2000 for each variable under the assumption that the composition of the U.S. cancer population is significantly different between 2000 and recent years.

### Analyses

We conducted all analyses using the R statistical software package version 4.2.1 [20]. To demonstrate the advantage of PSI in detecting distribution differences between large samples, we compared the PSI results to the results of Chi-square tests. We calculated the Chi-Square test scores for the comparisons we used to compute PSI scores. We adjusted the p-value using the Bonferroni approach for the Chi-square test due to multiple comparisons. For the comparisons showing significance in the Chi-Square test, we used Cramér's V to estimate the size of differences between the samples [21]. In this study, we consider a Cramér's V score < = 0.2 for a small effect size, a score > 0.2 and < = 0.6 representing a moderate effect size, and a score > 0.6 for a large effect size [22].

## Results

We present a yearly summary of U.S. cancer population counts and percentages by age, sex, and cancer site group in Table 1.

**Table 1:** Yearly U.S. cancer population characteristics

Year	2000			2015			2016			2017			2018			2019			2020		
Group	count	%	Crude rate	count	%	Crude rate	count	%	Crude rate	count	%	Crude rate	count	%	Crude rate	count	%	Crude rate	count	%	Crude rate
Age group																					
15–19	260	0.10%	4.8	327	0.10%	5.8	338	0.10%	6	289	0.10%	5.1	307	0.10%	5.5	321	0.10%	5.7	310	0.10%	5.5
20–24	663	0.30%	12.8	818	0.30%	13.3	888	0.30%	14.7	816	0.30%	13.7	802	0.30%	13.7	812	0.30%	14	751	0.30%	13
25–29	1,408	0.60%	26.4	1,830	0.60%	29.7	1,845	0.60%	29.2	1,840	0.60%	28.5	1,734	0.60%	26.7	1,754	0.60%	27.1	1,576	0.60%	24.8
30–34	2,551	1.10%	44.7	3,154	1.10%	52.9	3,376	1.20%	56.1	3,258	1.10%	53.9	3,263	1.10%	53.7	3,391	1.10%	55	3,208	1.20%	51.2
35–39	4,989	2.10%	81.2	4,913	1.70%	87.9	5,039	1.70%	88.3	5,131	1.70%	88.3	5,215	1.70%	88.3	5,377	1.70%	90.2	5,019	1.80%	84.1
40–44	8,726	3.60%	145.9	8,212	2.90%	149.8	7,948	2.80%	147.9	8,060	2.70%	150.5	8,183	2.70%	152.2	8,564	2.80%	157.8	7,839	2.80%	142.1
45–49	13,039	5.40%	246	13,487	4.80%	240.5	13,206	4.60%	234.1	13,574	4.60%	241.1	13,375	4.50%	240	13,562	4.40%	247.6	12,302	4.40%	229.6
50–54	18,966	7.80%	410	23,075	8.10%	394	22,410	7.80%	389.7	21,845	7.40%	387.3	21,140	7.10%	382.2	20,996	6.80%	385.9	18,681	6.70%	344.3
55–59	23,800	9.80%	690.3	31,918	11.20%	568.2	32,388	11.20%	571.2	32,540	11.00%	573.2	31,763	10.70%	559.8	32,569	10.60%	575.3	28,342	10.20%	507.4
60–64	27,699	11.40%	1029.2	39,638	14.00%	821	40,104	13.90%	810.9	42,007	14.20%	829.1	42,480	14.30%	824.2	43,695	14.20%	838.8	38,976	14.10%	740.6
65–69	32,891	13.60%	1423.5	45,892	16.20%	1147.9	47,686	16.50%	1141.5	48,664	16.40%	1164.4	48,336	16.20%	1138.4	50,314	16.30%	1160.2	45,153	16.30%	1018.7
70–74	35,945	14.80%	1693.8	38,254	13.50%	1361.7	39,901	13.80%	1373.2	43,173	14.60%	1368.9	44,597	15.00%	1352.6	47,002	15.30%	1362.4	42,869	15.50%	1191.6
75–79	33,045	13.60%	1832	29,299	10.30%	1482.4	30,180	10.50%	1480.6	31,373	10.60%	1477.8	33,207	11.10%	1477.8	35,108	11.40%	1497.4	32,284	11.70%	1328.5
80–84	22,046	9.10%	1837.7	21,711	7.70%	1532.3	21,523	7.50%	1500.3	22,087	7.50%	1516	22,374	7.50%	1495.7	23,029	7.50%	1493.8	20,778	7.50%	1316.2
85-	16,704	6.90%	1637	21,237	7.50%	1360.3	21,508	7.50%	1350.8	21,179	7.20%	1312.7	21,297	7.10%	1305.2	21,336	6.90%	1292.6	18,841	6.80%	1131.7
Sex																					
Male	122,045	50.30%	428.3	134,104	47.30%	398.3	137,671	47.70%	405.3	142,201	48.10%	415.5	143,046	48.00%	415.5	149,010	48.40%	430.7	133,557	48.20%	384.7
Female	120,687	49.70%	404.7	149,661	52.70%	427.4	150,669	52.30%	426.6	153,635	51.90%	432	155,027	52.00%	433.4	158,820	51.60%	441.9	143,372	51.80%	397.6
Cancer group																					
Breast	51,078	21.00%	87.6	64,137	22.60%	93.4	64,335	22.30%	92.9	66,014	22.30%	94.6	67,371	22.60%	96.0	69,369	22.50%	98.3	63,066	22.80%	89.1
Colorectal	111,303	45.90%	190.9	108,870	38.40%	158.5	109,632	38.00%	158.2	108,315	36.60%	155.2	109,503	36.70%	156.0	111,858	36.30%	158.6	99,771	36.00%	141.0
Genitourinary	200,929	82.80%	344.6	235,916	83.10%	343.5	244,809	84.90%	353.3	255,629	86.40%	366.3	258,067	86.60%	367.6	268,403	87.20%	380.5	245,058	88.50%	346.2
Lung	45,748	18.80%	78.5	49,469	17.40%	72.0	49,489	17.20%	71.4	49,893	16.90%	71.5	49,167	16.50%	70.0	50,099	16.30%	71.0	44,280	16.00%	62.6
Melanoma	12,712	5.20%	21.8	22,842	8.00%	33.3	22,797	7.90%	32.9	23,374	7.90%	33.5	23,508	7.90%	33.5	24,564	8.00%	34.8	20,948	7.60%	29.6

Note: Crude rates are per 100,000

**Fig. 1:**
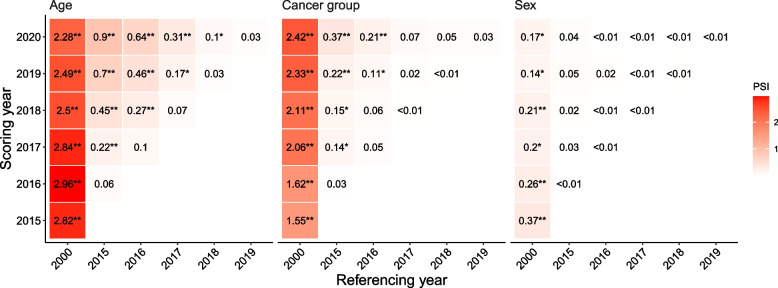
Population stability Index for year comparison pairs by age, sex, and cancer site. Note: ** represent PSI >= 0.25; * indicates PSI >= 0.1 and < 0.25

PSI scores comparing age, sex, and cancer site distribution between years ranged widely from 2.96 to less than 0.01 (Fig. 1). PSI scores indicate moderate to significant differences in cancer population characteristics between 2000 and all other included years. PSI scores are less likely to reach the moderate or significant difference thresholds when the referencing and scoring years are closer.

The largest PSI was 2.96 for the age group comparison between 2016 and 2000. Further investigation of the composition of the PSI score reveals that there were notably more cancer individuals in the age groups of 60–64, 65–69, and 75–79 years in 2016 (Table 2). We included the PSI calculation processes for all comparisons in the Online Appendix.

**Table 2:** PSI comparing differences in age distribution for U.S. cancer population between year 2000 and 2016

Age group	2000	2016	Difference	Natural logarithm	PSI	Total PSI
15–19	0.11	0.12	−0.01	−0.09	0	2.96
20–24	0.27	0.31	−0.03	−0.12	0	
25–29	0.58	0.64	−0.06	−0.1	0.01	
30–34	1.05	1.17	−0.12	−0.11	0.01	
35–39	2.06	1.75	0.31	0.16	0.05	
40–44	3.59	2.76	0.84	0.27	0.22	
45–49	5.37	4.58	0.79	0.16	0.13	
50–54	7.81	7.77	0.04	0.01	0	
55–59	9.81	11.23	−1.43	−0.14	0.19	
60–64	11.41	13.91	−2.5	−0.2	0.49	
65–69	13.55	16.54	−2.99	−0.2	0.6	
70–74	14.81	13.84	0.97	0.07	0.07	
75–79	13.61	10.47	3.15	0.26	0.83	
80–84	9.08	7.46	1.62	0.2	0.32	
85+	6.88	7.46	−0.58	−0.08	0.05	

**Fig. 2:**
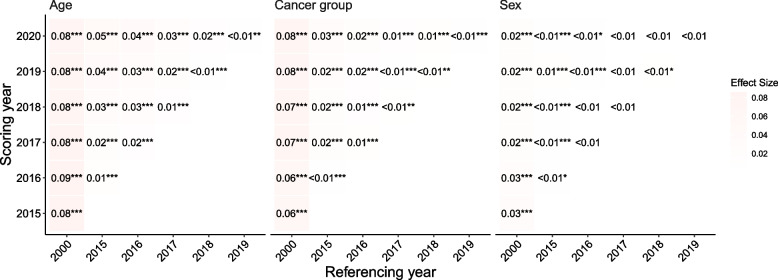
Cramér's V effect size scores for year comparison pairs by age, sex, and cancer site. Note: *** represent *p*-value <0.000047; ** indicates *p*-value <0.00047; * means *p*-value <0.0023

We included the Chi-Square test and effect size results in Fig. 2. The Chi-Square tests showed significance for most comparisons. On the other hand, Cramér's V scores for the comparisons with significant Chi-Square scores revealed that the effect sizes were all small, ranging from less than 0.01 to 0.09.

## Discussion

Quantitatively assessing differences in research samples provides a means to accurately describe sample representativeness for observational studies and allow proper evaluation and informed use of scientific evidence. Many retrospective cohort studies in healthcare leverage electronic health record (EHR) data and discover knowledge using massive data with much larger sample sizes than before. However, traditional tools, such as Pearson's Chi-Square test and Student-T test, for the examination of sample differences have too much power to discard subtle differences that may not be clinically meaningful when the sample size increases to over a thousand people [6, 10]. In this study, we examined the capacity of the population stability index (PSI) to detect sample differences and compared the PSI results to the Chi-Square test results. Our results suggest that PSI can detect differences in the distribution of given variables between two large samples and estimate the differences unaffected by the overpowering issue.

Our PSI results suggested that the U.S. cancer population after 2015 significantly differs from the population in 2000 in terms of sex, age, and cancer groups, but the differences between any two consecutive years are ignorable, aligning with previous epidemiology surveillance reports [23]. On the other hand, the traditional approaches showed significance in most comparisons after the application of an abnormally aggressive p-value adjustment with tiny V scores (< 0.01), indicating ignorable differences. This may be problematic for comparisons between 2000 and recent years, such as 2015, as evidence has shown that the populations between the years are different [24]. Our findings suggest that PSI provides a better estimate of sample differences when the sample size is large with an inevitable overpowering issue.

The PSI scores are the summation of the score for each category of the variate used to example sample differences. The breakdown scores provide additional information to enable the identification of categories that contribute to the sample differences. The example we provided in the results section demonstrated that use of PSI to examine age group differences in U.S. cancer populations in 2000 and 2016 enables the findings that the U.S. cancer population is notably older than before. Although the age group comparison between 2000 and 2016 may not provide meaningful information, the analysis can be utilized in other scenarios to guide further investigation or analysis approach adjustment. Example scenarios include assessing cancer-type differences between immunotherapy patients with and without the development of adverse events using population-based data, comparing sample differences between control and intervention groups for large, multi-institutional clinical trials, and evaluating whether a machine learning model is applicable to a population.

Researchers have argued that statistical approaches for hypothesis testing using p-value should not be used to assess sample differences between large datasets without adjustment [25]. The rationale behind using the Chi-square test was to emphasize its limitation and to highlight that PSI can be used for sample representativeness estimation in a big data context. We were not able to compare PSI and other sample representativeness metrics designed for large sample comparisons due to the lack of suitable large data access. However, this limitation highlighted a notable advantage of PSI that it can be computed using aggregated data. Most population databases, such as SEER, International Agency for Research on Cancer (IARC), and other disease-specific registry databases, are only available in an aggregated format without further permission. Therefore, few research teams can access and leverage raw population data to evaluate the representativeness of their samples using assessment metrics that require raw data, such as R indicators and standardized mean difference (SMD) approaches [7–9]. Our findings suggested that PSI can arguably be an alternative to those representativeness metrics developed by epidemiologists when raw population data are unavailable. When raw population data are available, PSI can complement the representativeness metrics, providing overall representativeness scores, as PSI enables information about differences in the category distribution of a variable in two samples.

Given the popularity of PSI in the AI and ML industry for monitoring feature drift in data, PSI has been widely implemented in many ML tool kits, such as Azure AI, Evidently AI, and Neptune AI. The current implementation of PSI in these ML packages that require raw continuous data can be improved by allowing the use of aggregated data to enable broader research teams to leverage publicly available population data for sample representativeness assessment. These AI tool kits also provide other sample similarity metrics, such as Kullback–Leibler (KL) divergence and Jensen-Shannon (JS) distance, which also require raw data. These metrics are similar to PSI estimating sample similarity based on differences in variable distributions [14, 26]. Further research is needed to compare PSI and other distance-based metrics to correlate the results of these metrics.

It is also essential to discuss the limitations of PSI to examine sample differences. First, PSI requires the variable of interest to be categorical and needs numerical variables to be binned before score calculation. Thus, information loss may happen when discretizing numeric variables [27], and bin size selection can determine PSI scores [28], similar to plotting a histogram of a numeric variable of two samples. Second, although there is wide use of PSI in industry and researchers have tried to define the statistical property of the PSI score [28, 29], little discussion on the metric and the score interpretation is in the literature body. Further, PSI cannot detect selection bias if the same selection bias exists in both samples.

The PSI score was designed for univariant comparisons, and thus, multivariant conditions were not considered. It was mostly used to detect data drifting in the AI/ML industry, with the primary goal of detecting notable changes in sample distribution for any variables [28]. It is possible to concatenate multiple variables into a single variable for each individual in the sample after binning them and calculating PSI scores for the concatenated variable. In this way, multiple variables were considered at once and may provide further information about the sample representativeness. However, this approach would require access to raw data and thus could not be conducted in this analysis. Future experiments are needed to explore the use of PSI for multivariate analyses.With the limitations of PSI, we suggest the PSI is a good alternative for population representativeness evaluation when raw data is not available for other approaches, such as R indicators, and when the sample size is large that traditional statistics inevitably capture a significant difference in samples with a clinically ignorable effect size.

This study has limitations. First, due to the use of SEER data, all data accessible to us were aggregated and categorical. Therefore, we could not apply PSI to a numeric variable and compare its results to the Student T-test or ANOVA results or compare PSI to R-indicators. Further, the dataset we used contains data from over 240,000 patients per year. This sample size may be greater than common big data studies with sample sizes ranging from 1,000 to 15,000. It is unclear whether the issues with inflated power of traditional approaches persists in such a sample size, thus requiring further investigation. There is also a need to examine the applicability of PSI in sample difference detection using data with a sample size similar to general healthcare research using big data.

## Conclusions

Sample representativeness is a key determinant of the generalizability and applicability of study findings. In this study, we examined the use of PSI to capture differences in large samples and compared its results to the traditional statistics. Our findings suggest that PSI can be used to examine sample differences in healthcare studies leveraging big data. Further research is needed to compare the PSI to other sample representativeness metrics and correlate their results to enable comparable data. Further implementation allowing the use of aggregated data for PSI calculation will enable research teams to use the metric using aggregated population-based datasets.

## Supplementary Information

Additional file 1.

## Data Availability

All data used in this study are publicly available from the Surveillance Epidemiology and End Results (SEER) Program. We published all our analysis data in this manuscript.

## Abbreviations

AI: Artificial intelligence
HER: Electronic heath record
IARC: International Agency for Research on Cancer
JS: Jensen-Shannon distance
KL: Kullback–Leibler divergence
ML: Machine learning
PSI: Population Index Stability
SEER: Surveillance, Epidemiology, and End Results
SMD: Standardized mean difference
