# Supplementary material for: Assessing the representativeness of large medical data using population stability index
Source: BMC Med Res Methodol. 2025 Feb 21;25:44. doi: 10.1186/s12874-025-02474-9 (PMC11844046; doi:10.1186/s12874-025-02474-9)
Supplement: Supplementary file 1 — Additional file 1. [file 12874_2025_2474_MOESM1_ESM.docx]

Online Appendix

Assessing the representativeness of large, opportunistic data using population stability index

Sheng-Chieh Lu, Wenye Song, Andre Pfob, Chris Gibbons

eTable 1. U.S. cancer population differences by age, sex, and cancer groups between year 2000 and 2015.

| Groups | 2000 | 2015 | Difference | Natural logarithm | PSI | Total PSI | Chi-square | Effect size |
| --- | --- | --- | --- | --- | --- | --- | --- | --- |
| **Age** | | | | | | |  |  |
| 15-19 | 0.11 | 0.12 | -0.01 | -0.07 | <0.01 | 2.82 | ꭓ^2^ = 3678.48, df = 14, *p* < 0.0001 | 0.08 |
| 20-24 | 0.27 | 0.29 | -0.02 | -0.05 | <0.01 |  |  |  |
| 25-29 | 0.58 | 0.64 | -0.06 | -0.11 | 0.01 |  |  |  |
| 30-34 | 1.05 | 1.11 | -0.06 | -0.06 | <0.01 |  |  |  |
| 35-39 | 2.06 | 1.73 | 0.32 | 0.17 | 0.06 |  |  |  |
| 40-44 | 3.59 | 2.89 | 0.70 | 0.22 | 0.15 |  |  |  |
| 45-49 | 5.37 | 4.75 | 0.62 | 0.12 | 0.08 |  |  |  |
| 50-54 | 7.81 | 8.13 | -0.32 | -0.04 | 0.01 |  |  |  |
| 55-59 | 9.81 | 11.25 | -1.44 | -0.14 | 0.20 |  |  |  |
| 60-64 | 11.41 | 13.97 | -2.56 | -0.20 | 0.52 |  |  |  |
| 65-69 | 13.55 | 16.17 | -2.62 | -0.18 | 0.46 |  |  |  |
| 70-74 | 14.81 | 13.48 | 1.33 | 0.09 | 0.12 |  |  |  |
| 75-79 | 13.61 | 10.33 | 3.29 | 0.28 | 0.91 |  |  |  |
| 80-84 | 9.08 | 7.65 | 1.43 | 0.17 | 0.25 |  |  |  |
| 85+ | 6.88 | 7.48 | -0.60 | -0.08 | 0.05 |  |  |  |
| **Sex** | | | | | | |  |  |
| Male | 49.72 | 52.74 | -3.02 | -0.06 | 0.18 | 0.37 | ꭓ^2^ = 477.78, df = 1, *p* < 0.0001 | 0.03 |
| Female | 50.28 | 47.26 | 3.02 | 0.06 | 0.19 |  |  |  |
| **Cancer group** | | | | | | |  |  |
| Breast | 12.11 | 13.33 | -1.22 | -0.10 | 0.12 | 1.55 | ꭓ^2^ = 3439.94, df = 3, p < 0.0001 | 0.06 |
| Colorectal | 26.39 | 22.62 | 3.77 | 0.15 | 0.58 |  |  |  |
| Genitourinary | 47.64 | 49.02 | -1.38 | -0.03 | 0.04 |  |  |  |
| Lung | 10.85 | 10.28 | 0.57 | 0.05 | 0.03 |  |  |  |
| Melanoma | 3.01 | 4.75 | -1.73 | -0.45 | 0.79 |  |  |  |

eTable 2. U.S. cancer population differences by age, sex, and cancer groups between year 2000 and 2016.

| Groups | 2000 | 2016 | Difference | Natural logarithm | PSI | Total PSI | Chi-square | Effect size |
| --- | --- | --- | --- | --- | --- | --- | --- | --- |
| **Age** | | | | | | |  |  |
| 15-19 | 0.11 | 0.12 | -0.01 | -0.09 | <0.01 | 2.96 | ꭓ^2^ = 3897.76, df = 14, *p* < 0.0001 | 0.09 |
| 20-24 | 0.27 | 0.31 | -0.03 | -0.12 | <0.01 |  |  |  |
| 25-29 | 0.58 | 0.64 | -0.06 | -0.10 | 0.01 |  |  |  |
| 30-34 | 1.05 | 1.17 | -0.12 | -0.11 | 0.01 |  |  |  |
| 35-39 | 2.06 | 1.75 | 0.31 | 0.16 | 0.05 |  |  |  |
| 40-44 | 3.59 | 2.76 | 0.84 | 0.27 | 0.22 |  |  |  |
| 45-49 | 5.37 | 4.58 | 0.79 | 0.16 | 0.13 |  |  |  |
| 50-54 | 7.81 | 7.77 | 0.04 | 0.01 | <0.01 |  |  |  |
| 55-59 | 9.81 | 11.23 | -1.43 | -0.14 | 0.19 |  |  |  |
| 60-64 | 11.41 | 13.91 | -2.50 | -0.20 | 0.49 |  |  |  |
| 65-69 | 13.55 | 16.54 | -2.99 | -0.20 | 0.60 |  |  |  |
| 70-74 | 14.81 | 13.84 | 0.97 | 0.07 | 0.07 |  |  |  |
| 75-79 | 13.61 | 10.47 | 3.15 | 0.26 | 0.83 |  |  |  |
| 80-84 | 9.08 | 7.46 | 1.62 | 0.20 | 0.32 |  |  |  |
| 85+ | 6.88 | 7.46 | -0.58 | -0.08 | 0.05 |  |  |  |
| **Sex** | | | | | | |  |  |
| Male | 49.72 | 52.25 | -2.53 | -0.05 | 0.13 | 0.26 | ꭓ^2^ = 338.47, df = 1, *p* < 0.0001 | 0.03 |
| Female | 50.28 | 47.75 | 2.53 | 0.05 | 0.13 |  |  |  |
| **Cancer group** | | | | | | |  |  |
| Breast | 12.11 | 13.10 | -0.99 | -0.08 | 0.08 | 1.62 | ꭓ^2^ = 3627.36, df = 3, *p* < 0.0001 | 0.06 |
| Colorectal | 26.39 | 22.33 | 4.06 | 0.17 | 0.68 |  |  |  |
| Genitourinary | 47.64 | 49.85 | -2.21 | -0.05 | 0.10 |  |  |  |
| Lung | 10.85 | 10.08 | 0.77 | 0.07 | 0.06 |  |  |  |
| Melanoma | 3.01 | 4.64 | -1.63 | -0.43 | 0.70 |  |  |  |

eTable 3. U.S. cancer population differences by age, sex, and cancer groups between year 2000 and 2017.

| Groups | 2000 | 2017 | Difference | Natural logarithm | PSI | Total PSI | Chi-square | Effect size |
| --- | --- | --- | --- | --- | --- | --- | --- | --- |
| **Age** | | | | | | |  |  |
| 15-19 | 0.11 | 0.10 | 0.01 | 0.09 | <0.01 | 2.84 | ꭓ^2^ = 3779.36,  df = 14,  *p* < 0.0001 | 0.08 |
| 20-24 | 0.27 | 0.28 | <0.01 | -0.01 | <0.01 |  |  |  |
| 25-29 | 0.58 | 0.62 | -0.04 | -0.07 | <0.01 |  |  |  |
| 30-34 | 1.05 | 1.10 | -0.05 | -0.05 | <0.01 |  |  |  |
| 35-39 | 2.06 | 1.73 | 0.32 | 0.17 | 0.05 |  |  |  |
| 40-44 | 3.59 | 2.72 | 0.87 | 0.28 | 0.24 |  |  |  |
| 45-49 | 5.37 | 4.59 | 0.78 | 0.16 | 0.12 |  |  |  |
| 50-54 | 7.81 | 7.38 | 0.43 | 0.06 | 0.02 |  |  |  |
| 55-59 | 9.81 | 11.00 | -1.19 | -0.11 | 0.14 |  |  |  |
| 60-64 | 11.41 | 14.20 | -2.79 | -0.22 | 0.61 |  |  |  |
| 65-69 | 13.55 | 16.45 | -2.90 | -0.19 | 0.56 |  |  |  |
| 70-74 | 14.81 | 14.59 | 0.21 | 0.01 | <0.01 |  |  |  |
| 75-79 | 13.61 | 10.60 | 3.01 | 0.25 | 0.75 |  |  |  |
| 80-84 | 9.08 | 7.47 | 1.62 | 0.20 | 0.32 |  |  |  |
| 85+ | 6.88 | 7.16 | -0.28 | -0.04 | 0.01 |  |  |  |
| **Sex** | | | | | | |  |  |
| Male | 49.72 | 51.93 | -2.21 | -0.04 | 0.10 | 0.20 | ꭓ^2^ = 261.01,  df = 1,  *p* < 0.0001 | 0.02 |
| Female | 50.28 | 48.07 | 2.21 | 0.04 | 0.10 |  |  |  |
| **Cancer group** | | | | | | |  |  |
| Breast | 12.11 | 13.12 | -1.01 | -0.08 | 0.08 | 2.06 | ꭓ^2^ = 4692.75,  df = 3,  *p* < 0.0001 | 0.07 |
| Colorectal | 26.39 | 21.52 | 4.87 | 0.20 | 0.99 | 2.06 |  |  |
| Genitourinary | 47.64 | 50.80 | -3.16 | -0.06 | 0.20 | 2.06 |  |  |
| Lung | 10.85 | 9.91 | 0.93 | 0.09 | 0.08 | 2.06 |  |  |
| Melanoma | 3.01 | 4.64 | -1.63 | -0.43 | 0.71 | 2.06 |  |  |

eTable 4. U.S. cancer population differences by age, sex, and cancer groups between year 2000 and 2018.

| Groups | 2000 | 2018 | Difference | Natural logarithm | PSI | Total PSI | Chi-square | Effect size |
| --- | --- | --- | --- | --- | --- | --- | --- | --- |
| **Age** | | | | | | |  |  |
| 15-19 | 0.11 | 0.10 | <0.01 | 0.04 | <0.01 | 2.50 | ꭓ^2^ = 3333.02,  df = 14,  *p* < 0.0001 | 0.08 |
| 20-24 | 0.27 | 0.27 | <0.01 | 0.02 | <0.01 |  |  |  |
| 25-29 | 0.58 | 0.58 | <0.01 | <0.01 | <0.01 |  |  |  |
| 30-34 | 1.05 | 1.09 | -0.04 | -0.04 | <0.01 |  |  |  |
| 35-39 | 2.06 | 1.75 | 0.31 | 0.16 | 0.05 |  |  |  |
| 40-44 | 3.59 | 2.75 | 0.85 | 0.27 | 0.23 |  |  |  |
| 45-49 | 5.37 | 4.49 | 0.88 | 0.18 | 0.16 |  |  |  |
| 50-54 | 7.81 | 7.09 | 0.72 | 0.10 | 0.07 |  |  |  |
| 55-59 | 9.81 | 10.66 | -0.85 | -0.08 | 0.07 |  |  |  |
| 60-64 | 11.41 | 14.25 | -2.84 | -0.22 | 0.63 |  |  |  |
| 65-69 | 13.55 | 16.22 | -2.67 | -0.18 | 0.48 |  |  |  |
| 70-74 | 14.81 | 14.96 | -0.15 | -0.01 | <0.01 |  |  |  |
| 75-79 | 13.61 | 11.14 | 2.47 | 0.20 | 0.50 |  |  |  |
| 80-84 | 9.08 | 7.51 | 1.58 | 0.19 | 0.30 |  |  |  |
| 85+ | 6.88 | 7.14 | -0.26 | -0.04 | 0.01 |  |  |  |
| **Sex** | | | | | | |  |  |
| Male | 49.72 | 52.01 | -2.29 | -0.05 | 0.10 | 0.21 | ꭓ^2^ = 280.52,  df = 1,  *p* < 0.0001 | 0.02 |
| Female | 50.28 | 47.99 | 2.29 | 0.05 | 0.11 | 0.21 |  |  |
| **Cancer group** | | | | | | |  |  |
| Breast | 12.11 | 13.27 | -1.16 | -0.09 | 0.11 | 2.11 | ꭓ^2^ = 4821.39,  df = 3,  *p* < 0.0001 | 0.07 |
| Colorectal | 26.39 | 21.57 | 4.82 | 0.20 | 0.97 | 2.11 |  |  |
| Genitourinary | 47.64 | 50.84 | -3.20 | -0.07 | 0.21 | 2.11 |  |  |
| Lung | 10.85 | 9.69 | 1.16 | 0.11 | 0.13 | 2.11 |  |  |
| Melanoma | 3.01 | 4.63 | -1.62 | -0.43 | 0.69 | 2.11 |  |  |

eTable 5. U.S. cancer population differences by age, sex, and cancer groups between year 2000 and 2019.

| Groups | 2000 | 2019 | Difference | Natural logarithm | PSI | Total PSI | Chi-square | Effect size |
| --- | --- | --- | --- | --- | --- | --- | --- | --- |
| **Age** | | | | | | |  |  |
| 15-19 | 0.11 | 0.10 | <0.01 | 0.03 | <0.01 | 2.49 | ꭓ^2^ = 3375.83,  df = 14,  *p* < 0.0001 | 0.08 |
| 20-24 | 0.27 | 0.26 | 0.01 | 0.03 | <0.01 | 2.49 |  |  |
| 25-29 | 0.58 | 0.57 | 0.01 | 0.02 | <0.01 | 2.49 |  |  |
| 30-34 | 1.05 | 1.10 | -0.05 | -0.05 | <0.01 | 2.49 |  |  |
| 35-39 | 2.06 | 1.75 | 0.31 | 0.16 | 0.05 | 2.49 |  |  |
| 40-44 | 3.59 | 2.78 | 0.81 | 0.26 | 0.21 | 2.49 |  |  |
| 45-49 | 5.37 | 4.41 | 0.97 | 0.20 | 0.19 | 2.49 |  |  |
| 50-54 | 7.81 | 6.82 | 0.99 | 0.14 | 0.13 | 2.49 |  |  |
| 55-59 | 9.81 | 10.58 | -0.78 | -0.08 | 0.06 | 2.49 |  |  |
| 60-64 | 11.41 | 14.19 | -2.78 | -0.22 | 0.61 | 2.49 |  |  |
| 65-69 | 13.55 | 16.34 | -2.79 | -0.19 | 0.52 | 2.49 |  |  |
| 70-74 | 14.81 | 15.27 | -0.46 | -0.03 | 0.01 | 2.49 |  |  |
| 75-79 | 13.61 | 11.40 | 2.21 | 0.18 | 0.39 | 2.49 |  |  |
| 80-84 | 9.08 | 7.48 | 1.60 | 0.19 | 0.31 | 2.49 |  |  |
| 85+ | 6.88 | 6.93 | -0.05 | -0.01 | <0.01 | 2.49 |  |  |
| **Sex** | | | | | | |  |  |
| Male | 49.72 | 51.59 | -1.87 | -0.04 | 0.07 | 0.14 | ꭓ^2^ = 190.44,  df = 1,  *p* < 0.0001 | 0.02 |
| Female | 50.28 | 48.41 | 1.87 | 0.04 | 0.07 | 0.14 |  |  |
| **Cancer group** | | | | | | |  |  |
| Breast | 12.11 | 13.23 | -1.12 | -0.09 | 0.10 | 2.33 | ꭓ^2^ = 5398.58,  df = 3,  *p* < 0.0001 | 0.08 |
| Colorectal | 26.39 | 21.34 | 5.05 | 0.21 | 1.07 | 2.33 |  |  |
| Genitourinary | 47.64 | 51.19 | -3.55 | -0.07 | 0.26 | 2.33 |  |  |
| Lung | 10.85 | 9.56 | 1.29 | 0.13 | 0.16 | 2.33 |  |  |
| Melanoma | 3.01 | 4.69 | -1.67 | -0.44 | 0.74 | 2.33 |  |  |

eTable 6. U.S. cancer population differences by age, sex, and cancer groups between year 2000 and 2020.

| Groups | 2000 | 2020 | Difference | Natural logarithm | PSI | Total PSI | Chi-square | Effect size |
| --- | --- | --- | --- | --- | --- | --- | --- | --- |
| **Age** | | | | | | |  |  |
| 15-19 | 0.11 | 0.11 | <0.01 | -0.04 | <0.01 | 2.28 | ꭓ^2^ = 2940.51,  df = 14,  *p* < 0.0001 | 0.08 |
| 20-24 | 0.27 | 0.27 | <0.01 | 0.01 | <0.01 |  |  |  |
| 25-29 | 0.58 | 0.57 | 0.01 | 0.02 | <0.01 |  |  |  |
| 30-34 | 1.05 | 1.16 | -0.11 | -0.10 | 0.01 |  |  |  |
| 35-39 | 2.06 | 1.81 | 0.24 | 0.13 | 0.03 |  |  |  |
| 40-44 | 3.59 | 2.83 | 0.76 | 0.24 | 0.18 |  |  |  |
| 45-49 | 5.37 | 4.44 | 0.93 | 0.19 | 0.18 |  |  |  |
| 50-54 | 7.81 | 6.75 | 1.07 | 0.15 | 0.16 |  |  |  |
| 55-59 | 9.81 | 10.23 | -0.43 | -0.04 | 0.02 |  |  |  |
| 60-64 | 11.41 | 14.07 | -2.66 | -0.21 | 0.56 |  |  |  |
| 65-69 | 13.55 | 16.30 | -2.75 | -0.19 | 0.51 |  |  |  |
| 70-74 | 14.81 | 15.48 | -0.67 | -0.04 | 0.03 |  |  |  |
| 75-79 | 13.61 | 11.66 | 1.96 | 0.16 | 0.30 |  |  |  |
| 80-84 | 9.08 | 7.50 | 1.58 | 0.19 | 0.30 |  |  |  |
| 85+ | 6.88 | 6.80 | 0.08 | 0.01 | <0.01 |  |  |  |
| **Sex** | | | | | | |  |  |
| Male | 49.72 | 51.77 | -2.05 | -0.04 | 0.08 | 0.17 | ꭓ^2^ = 217.81,  df = 1,  *p* < 0.0001 | 0.02 |
| Female | 50.28 | 48.23 | 2.05 | 0.04 | 0.09 |  |  |  |
| **Cancer group** | | | | | | |  |  |
| Breast | 12.11 | 13.33 | -1.22 | -0.10 | 0.12 | 2.42 | ꭓ^2^ = 5365.46,  df = 3,  *p* < 0.0001 | 0.08 |
| Colorectal | 26.39 | 21.09 | 5.30 | 0.22 | 1.19 |  |  |  |
| Genitourinary | 47.64 | 51.80 | -4.16 | -0.08 | 0.35 |  |  |  |
| Lung | 10.85 | 9.36 | 1.49 | 0.15 | 0.22 |  |  |  |
| Melanoma | 3.01 | 4.43 | -1.41 | -0.38 | 0.54 |  |  |  |

eTable 7. U.S. cancer population differences by age, sex, and cancer groups between year 2015 and 2016.

| Groups | 2015 | 2016 | Difference | Natural logarithm | PSI | Total PSI | Chi-square | Effect size |
| --- | --- | --- | --- | --- | --- | --- | --- | --- |
| **Age** | | | | | | |  |  |
| 15-19 | 0.12 | 0.12 | <0.01 | -0.02 | <0.01 | 0.06 | ꭓ^2^ = 83.52,  df = 14,  *p* < 0.0001 | 0.01 |
| 20-24 | 0.29 | 0.31 | -0.02 | -0.07 | <0.01 |  |  |  |
| 25-29 | 0.64 | 0.64 | 0.01 | 0.01 | <0.01 |  |  |  |
| 30-34 | 1.11 | 1.17 | -0.06 | -0.05 | <0.01 |  |  |  |
| 35-39 | 1.73 | 1.75 | -0.02 | -0.01 | <0.01 |  |  |  |
| 40-44 | 2.89 | 2.76 | 0.14 | 0.05 | 0.01 |  |  |  |
| 45-49 | 4.75 | 4.58 | 0.17 | 0.04 | 0.01 |  |  |  |
| 50-54 | 8.13 | 7.77 | 0.36 | 0.05 | 0.02 |  |  |  |
| 55-59 | 11.25 | 11.23 | 0.02 | <0.01 | <0.01 |  |  |  |
| 60-64 | 13.97 | 13.91 | 0.06 | <0.01 | <0.01 |  |  |  |
| 65-69 | 16.17 | 16.54 | -0.37 | -0.02 | 0.01 |  |  |  |
| 70-74 | 13.48 | 13.84 | -0.36 | -0.03 | 0.01 |  |  |  |
| 75-79 | 10.33 | 10.47 | -0.14 | -0.01 | <0.01 |  |  |  |
| 80-84 | 7.65 | 7.46 | 0.19 | 0.02 | <0.01 |  |  |  |
| 85+ | 7.48 | 7.46 | 0.02 | <0.01 | <0.01 |  |  |  |
| **Sex** | | | | | | |  |  |
| Male | 52.74 | 52.25 | 0.49 | 0.01 | <0.01 | 0.01 | ꭓ^2^ = 13.60,  df = 1,  *p* = 0.0002 | <0.01 |
| Female | 47.26 | 47.75 | -0.49 | -0.01 | <0.01 |  |  |  |
| **Cancer group** | | | | | | |  |  |
| Breast | 13.33 | 13.10 | 0.23 | 0.02 | <0.01 | 0.03 | ꭓ^2^ = 68.19,  df = 3,  *p* < 0.0001 | 0.01 |
| Colorectal | 22.62 | 22.33 | 0.30 | 0.01 | <0.01 |  |  |  |
| Genitourinary | 49.02 | 49.85 | -0.83 | -0.02 | 0.01 |  |  |  |
| Lung | 10.28 | 10.08 | 0.20 | 0.02 | <0.01 |  |  |  |
| Melanoma | 4.75 | 4.64 | 0.10 | 0.02 | <0.01 |  |  |  |

eTable 8. U.S. cancer population differences by age, sex, and cancer groups between year 2015 and 2017.

| Groups | 2015 | 2017 | Difference | Natural logarithm | PSI | Total PSI | Chi-square | Effect size |
| --- | --- | --- | --- | --- | --- | --- | --- | --- |
| **Age** | | | | | | |  |  |
| 15-19 | 0.12 | 0.10 | 0.02 | 0.17 | <0.01 | 0.22 | ꭓ^2^=320.28,  df = 14,  p<0.0001 | 0.02 |
| 20-24 | 0.29 | 0.28 | 0.01 | 0.04 | <0.01 |  |  |  |
| 25-29 | 0.64 | 0.62 | 0.02 | 0.04 | <0.01 |  |  |  |
| 30-34 | 1.11 | 1.10 | 0.01 | 0.01 | <0.01 |  |  |  |
| 35-39 | 1.73 | 1.73 | <0.01 | <0.01 | <0.01 |  |  |  |
| 40-44 | 2.89 | 2.72 | 0.17 | 0.06 | 0.01 |  |  |  |
| 45-49 | 4.75 | 4.59 | 0.16 | 0.04 | 0.01 |  |  |  |
| 50-54 | 8.13 | 7.38 | 0.75 | 0.10 | 0.07 |  |  |  |
| 55-59 | 11.25 | 11.00 | 0.25 | 0.02 | 0.01 |  |  |  |
| 60-64 | 13.97 | 14.20 | -0.23 | -0.02 | <0.01 |  |  |  |
| 65-69 | 16.17 | 16.45 | -0.28 | -0.02 | <0.01 |  |  |  |
| 70-74 | 13.48 | 14.59 | -1.11 | -0.08 | 0.09 |  |  |  |
| 75-79 | 10.33 | 10.60 | -0.28 | -0.03 | 0.01 |  |  |  |
| 80-84 | 7.65 | 7.47 | 0.19 | 0.02 | <0.01 |  |  |  |
| 85+ | 7.48 | 7.16 | 0.32 | 0.04 | 0.01 |  |  |  |
| **Sex** | | | | | | |  |  |
| Male | 52.74 | 51.93 | 0.81 | 0.02 | 0.01 | 0.03 | ꭓ^2^=37.94,  df = 1,  p<0.0001 | 0.01 |
| Female | 47.26 | 48.07 | -0.81 | -0.02 | 0.01 |  |  |  |
| **Cancer group** | | | | | | |  |  |
| Breast | 13.33 | 13.12 | 0.21 | 0.02 | <0.01 | 0.14 | ꭓ^2^ = 335.92,  df = 3,  *p* < 0.0001 | 0.02 |
| Colorectal | 22.62 | 21.52 | 1.10 | 0.05 | 0.05 |  |  |  |
| Genitourinary | 49.02 | 50.80 | -1.78 | -0.04 | 0.06 |  |  |  |
| Lung | 10.28 | 9.91 | 0.36 | 0.04 | 0.01 |  |  |  |
| Melanoma | 4.75 | 4.64 | 0.10 | 0.02 | <0.01 |  |  |  |

eTable 9. U.S. cancer population differences by age, sex, and cancer groups between year 2015 and 2018.

| Groups | 2015 | 2018 | Difference | Natural logarithm | PSI | Total PSI | Chi-square | Effect size |
| --- | --- | --- | --- | --- | --- | --- | --- | --- |
| **Age** | | | | | | |  |  |
| 15-19 | 0.12 | 0.10 | 0.01 | 0.11 | <0.01 | 0.45 | ꭓ^2^ = 650.11,  df = 14,  *p* < 0.0001 | 0.03 |
| 20-24 | 0.29 | 0.27 | 0.02 | 0.07 | <0.01 |  |  |  |
| 25-29 | 0.64 | 0.58 | 0.06 | 0.10 | 0.01 |  |  |  |
| 30-34 | 1.11 | 1.09 | 0.02 | 0.02 | <0.01 |  |  |  |
| 35-39 | 1.73 | 1.75 | -0.02 | -0.01 | <0.01 |  |  |  |
| 40-44 | 2.89 | 2.75 | 0.15 | 0.05 | 0.01 |  |  |  |
| 45-49 | 4.75 | 4.49 | 0.27 | 0.06 | 0.02 |  |  |  |
| 50-54 | 8.13 | 7.09 | 1.04 | 0.14 | 0.14 |  |  |  |
| 55-59 | 11.25 | 10.66 | 0.59 | 0.05 | 0.03 |  |  |  |
| 60-64 | 13.97 | 14.25 | -0.28 | -0.02 | 0.01 |  |  |  |
| 65-69 | 16.17 | 16.22 | -0.04 | <0.01 | <0.01 |  |  |  |
| 70-74 | 13.48 | 14.96 | -1.48 | -0.10 | 0.15 |  |  |  |
| 75-79 | 10.33 | 11.14 | -0.82 | -0.08 | 0.06 |  |  |  |
| 80-84 | 7.65 | 7.51 | 0.14 | 0.02 | <0.01 |  |  |  |
| 85+ | 7.48 | 7.14 | 0.34 | 0.05 | 0.02 |  |  |  |
| **Sex** | | | | | | |  |  |
| Male | 52.74 | 52.01 | 0.73 | 0.01 | 0.01 | 0.02 | ꭓ^2^ = 31.15,  df = 1,  *p* < 0.0001 | 0.01 |
| Female | 47.26 | 47.99 | -0.73 | -0.02 | 0.01 |  |  |  |
| **Cancer group** | | | | | | |  |  |
| Breast | 13.33 | 13.27 | 0.06 | <0.01 | <0.01 | 0.15 | ꭓ^2^ = 381.56, df = 3,  *p* < 0.0001 | 0.02 |
| Colorectal | 22.62 | 21.57 | 1.05 | 0.05 | 0.05 |  |  |  |
| Genitourinary | 49.02 | 50.84 | -1.82 | -0.04 | 0.07 |  |  |  |
| Lung | 10.28 | 9.69 | 0.59 | 0.06 | 0.04 |  |  |  |
| Melanoma | 4.75 | 4.63 | 0.12 | 0.02 | <0.01 |  |  |  |

eTable 10. U.S. cancer population differences by age, sex, and cancer groups between year 2015 and 2019.

| Groups | 2015 | 2019 | Difference | Natural logarithm | PSI | Total PSI | Chi-square | Effect size |
| --- | --- | --- | --- | --- | --- | --- | --- | --- |
| **Age** | | | | | | |  |  |
| 15-19 | 0.12 | 0.10 | 0.01 | 0.10 | <0.01 | 0.70 | ꭓ^2^ = 1027.58,  df = 14,  *p* < 0.0001 | 0.04 |
| 20-24 | 0.29 | 0.26 | 0.02 | 0.09 | <0.01 |  |  |  |
| 25-29 | 0.64 | 0.57 | 0.08 | 0.12 | 0.01 |  |  |  |
| 30-34 | 1.11 | 1.10 | 0.01 | 0.01 | <0.01 |  |  |  |
| 35-39 | 1.73 | 1.75 | -0.02 | -0.01 | <0.01 |  |  |  |
| 40-44 | 2.89 | 2.78 | 0.11 | 0.04 | <0.01 |  |  |  |
| 45-49 | 4.75 | 4.41 | 0.35 | 0.08 | 0.03 |  |  |  |
| 50-54 | 8.13 | 6.82 | 1.31 | 0.18 | 0.23 |  |  |  |
| 55-59 | 11.25 | 10.58 | 0.67 | 0.06 | 0.04 |  |  |  |
| 60-64 | 13.97 | 14.19 | -0.23 | -0.02 | <0.01 |  |  |  |
| 65-69 | 16.17 | 16.34 | -0.17 | -0.01 | <0.01 |  |  |  |
| 70-74 | 13.48 | 15.27 | -1.79 | -0.12 | 0.22 |  |  |  |
| 75-79 | 10.33 | 11.40 | -1.08 | -0.10 | 0.11 |  |  |  |
| 80-84 | 7.65 | 7.48 | 0.17 | 0.02 | <0.01 |  |  |  |
| 85+ | 7.48 | 6.93 | 0.55 | 0.08 | 0.04 |  |  |  |
| **Sex** | | | | | | |  |  |
| Male | 52.74 | 51.59 | 1.15 | 0.02 | 0.03 | 0.05 | ꭓ^2^ = 77.90,  df = 1,  *p* < 0.0001 | 0.01 |
| Female | 47.26 | 48.41 | -1.15 | -0.02 | 0.03 |  |  |  |
| **Cancer group** | | | | | | |  |  |
| Breast | 13.33 | 13.23 | 0.10 | 0.01 | <0.01 | 0.22 | ꭓ^2^ = 561.90,  df = 3,  *p* < 0.0001 | 0.02 |
| Colorectal | 22.62 | 21.34 | 1.29 | 0.06 | 0.08 |  |  |  |
| Genitourinary | 49.02 | 51.19 | -2.17 | -0.04 | 0.09 |  |  |  |
| Lung | 10.28 | 9.56 | 0.72 | 0.07 | 0.05 |  |  |  |
| Melanoma | 4.75 | 4.69 | 0.06 | 0.01 | <0.01 |  |  |  |

eTable 11. U.S. cancer population differences by age, sex, and cancer groups between year 2015 and 2020.

| Groups | 2015 | 2020 | Difference | Natural logarithm | PSI | Total PSI | Chi-square | Effect size |
| --- | --- | --- | --- | --- | --- | --- | --- | --- |
| **Age** | | | | | | |  |  |
| 15-19 | 0.12 | 0.11 | 0.01 | 0.03 | <0.01 | 0.90 | ꭓ^2^ = 1260.93,  df = 14,  *p* < 0.0001 | 0.05 |
| 20-24 | 0.29 | 0.27 | 0.02 | 0.06 | <0.01 |  |  |  |
| 25-29 | 0.64 | 0.57 | 0.08 | 0.13 | 0.01 |  |  |  |
| 30-34 | 1.11 | 1.16 | -0.05 | -0.04 | <0.01 |  |  |  |
| 35-39 | 1.73 | 1.81 | -0.08 | -0.05 | <0.01 |  |  |  |
| 40-44 | 2.89 | 2.83 | 0.06 | 0.02 | <0.01 |  |  |  |
| 45-49 | 4.75 | 4.44 | 0.31 | 0.07 | 0.02 |  |  |  |
| 50-54 | 8.13 | 6.75 | 1.39 | 0.19 | 0.26 |  |  |  |
| 55-59 | 11.25 | 10.23 | 1.01 | 0.09 | 0.10 |  |  |  |
| 60-64 | 13.97 | 14.07 | -0.11 | -0.01 | <0.01 |  |  |  |
| 65-69 | 16.17 | 16.30 | -0.13 | -0.01 | <0.01 |  |  |  |
| 70-74 | 13.48 | 15.48 | -2.00 | -0.14 | 0.28 |  |  |  |
| 75-79 | 10.33 | 11.66 | -1.33 | -0.12 | 0.16 |  |  |  |
| 80-84 | 7.65 | 7.50 | 0.15 | 0.02 | <0.01 |  |  |  |
| 85+ | 7.48 | 6.80 | 0.68 | 0.10 | 0.06 |  |  |  |
| **Sex** | | | | | | |  |  |
| Male | 52.74 | 51.77 | 0.97 | 0.02 | 0.02 | 0.04 | ꭓ^2^ = 52.72,  df = 1,  *p* < 0.0001 | 0.01 |
| Female | 47.26 | 48.23 | -0.97 | -0.02 | 0.02 |  |  |  |
| **Cancer group** | | | | | | |  |  |
| Breast | 13.33 | 13.33 | <0.01 | <0.01 | <0.01 | 0.37 | ꭓ^2^ = 879.86,  df = 3,  *p* < 0.0001 | 0.03 |
| Colorectal | 22.62 | 21.09 | 1.54 | 0.07 | 0.11 |  |  |  |
| Genitourinary | 49.02 | 51.80 | -2.77 | -0.06 | 0.15 |  |  |  |
| Lung | 10.28 | 9.36 | 0.92 | 0.09 | 0.09 |  |  |  |
| Melanoma | 4.75 | 4.43 | 0.32 | 0.07 | 0.02 |  |  |  |

eTable 12. U.S. cancer population differences by age, sex, and cancer groups between year 2016 and 2017.

| Groups | 2016 | 2017 | Difference | Natural logarithm | PSI | Total PSI | Chi-square | Effect size |
| --- | --- | --- | --- | --- | --- | --- | --- | --- |
| **Age** | | | | | | |  |  |
| 15-19 | 0.12 | 0.10 | 0.02 | 0.18 | <0.01 | 0.10 | ꭓ^2^ = 142.91,  df = 14,  *p* < 0.0001 | 0.02 |
| 20-24 | 0.31 | 0.28 | 0.03 | 0.11 | <0.01 |  |  |  |
| 25-29 | 0.64 | 0.62 | 0.02 | 0.03 | <0.01 |  |  |  |
| 30-34 | 1.17 | 1.10 | 0.07 | 0.06 | <0.01 |  |  |  |
| 35-39 | 1.75 | 1.73 | 0.01 | 0.01 | <0.01 |  |  |  |
| 40-44 | 2.76 | 2.72 | 0.03 | 0.01 | <0.01 |  |  |  |
| 45-49 | 4.58 | 4.59 | -0.01 | <0.01 | <0.01 |  |  |  |
| 50-54 | 7.77 | 7.38 | 0.39 | 0.05 | 0.02 |  |  |  |
| 55-59 | 11.23 | 11.00 | 0.23 | 0.02 | <0.01 |  |  |  |
| 60-64 | 13.91 | 14.20 | -0.29 | -0.02 | 0.01 |  |  |  |
| 65-69 | 16.54 | 16.45 | 0.09 | 0.01 | <0.01 |  |  |  |
| 70-74 | 13.84 | 14.59 | -0.76 | -0.05 | 0.04 |  |  |  |
| 75-79 | 10.47 | 10.60 | -0.14 | -0.01 | <0.01 |  |  |  |
| 80-84 | 7.46 | 7.47 | <0.01 | <0.01 | <0.01 |  |  |  |
| 85+ | 7.46 | 7.16 | 0.30 | 0.04 | 0.01 |  |  |  |
| **Sex** | | | | | | |  |  |
| Male | 52.25 | 51.93 | 0.32 | 0.01 | <0.01 | <0.01 | ꭓ^2^ = 6.03,  df = 1,  *p* = 0.0140 | <0.01 |
| Female | 47.75 | 48.07 | -0.32 | -0.01 | <0.01 |  |  |  |
| **Cancer group** | | | | | | |  |  |
| Breast | 13.10 | 13.12 | -0.02 | <0.01 | <0.01 | 0.05 | ꭓ^2^ = 123.61,  df = 3,  *p* < 0.0001 | 0.01 |
| Colorectal | 22.33 | 21.52 | 0.80 | 0.04 | 0.03 |  |  |  |
| Genitourinary | 49.85 | 50.80 | -0.95 | -0.02 | 0.02 |  |  |  |
| Lung | 10.08 | 9.91 | 0.16 | 0.02 | <0.01 |  |  |  |
| Melanoma | 4.64 | 4.64 | <0.01 | <0.01 | <0.01 |  |  |  |

eTable 13. U.S. cancer population differences by age, sex, and cancer groups between year 2016 and 2018.

| Groups | 2016 | 2018 | Difference | Natural logarithm | PSI | Total PSI | Chi-square | Effect size |
| --- | --- | --- | --- | --- | --- | --- | --- | --- |
| **Age** | | | | | | |  |  |
| 15-19 | 0.12 | 0.10 | 0.01 | 0.13 | <0.01 | 0.27 | ꭓ^2^ = 396.27,  df = 14,  *p* < 0.0001 | 0.03 |
| 20-24 | 0.31 | 0.27 | 0.04 | 0.14 | 0.01 |  |  |  |
| 25-29 | 0.64 | 0.58 | 0.06 | 0.10 | 0.01 |  |  |  |
| 30-34 | 1.17 | 1.09 | 0.08 | 0.07 | 0.01 |  |  |  |
| 35-39 | 1.75 | 1.75 | <0.01 | <0.01 | <0.01 |  |  |  |
| 40-44 | 2.76 | 2.75 | 0.01 | <0.01 | <0.01 |  |  |  |
| 45-49 | 4.58 | 4.49 | 0.09 | 0.02 | <0.01 |  |  |  |
| 50-54 | 7.77 | 7.09 | 0.68 | 0.09 | 0.06 |  |  |  |
| 55-59 | 11.23 | 10.66 | 0.58 | 0.05 | 0.03 |  |  |  |
| 60-64 | 13.91 | 14.25 | -0.34 | -0.02 | 0.01 |  |  |  |
| 65-69 | 16.54 | 16.22 | 0.32 | 0.02 | 0.01 |  |  |  |
| 70-74 | 13.84 | 14.96 | -1.12 | -0.08 | 0.09 |  |  |  |
| 75-79 | 10.47 | 11.14 | -0.67 | -0.06 | 0.04 |  |  |  |
| 80-84 | 7.46 | 7.51 | -0.04 | -0.01 | <0.01 |  |  |  |
| 85+ | 7.46 | 7.14 | 0.31 | 0.04 | 0.01 |  |  |  |
| **Sex** | | | | | | |  |  |
| Male | 52.25 | 52.01 | 0.24 | <0.01 | <0.01 | <0.01 | ꭓ^2^ = 3.49,  df = 1,  *p* = 0.617 | NA |
| Female | 47.75 | 47.99 | -0.24 | -0.01 | <0.01 |  |  |  |
| **Cancer group** | | | | | | |  |  |
| Breast | 13.10 | 13.27 | -0.17 | -0.01 | <0.01 | 0.06 | ꭓ^2^ = 157.21,  df = 3,  *p* < 0.0001 | 0.01 |
| Colorectal | 22.33 | 21.57 | 0.75 | 0.03 | 0.03 |  |  |  |
| Genitourinary | 49.85 | 50.84 | -0.99 | -0.02 | 0.02 |  |  |  |
| Lung | 10.08 | 9.69 | 0.39 | 0.04 | 0.02 |  |  |  |
| Melanoma | 4.64 | 4.63 | 0.01 | <0.01 | <0.01 |  |  |  |

eTable 14. U.S. cancer population differences by age, sex, and cancer groups between year 2016 and 2019.

| Groups | 2016 | 2019 | Difference | Natural logarithm | PSI | Total PSI | Chi-square | Effect size |
| --- | --- | --- | --- | --- | --- | --- | --- | --- |
| **Age** | | | | | | |  |  |
| 15-19 | 0.12 | 0.10 | 0.02 | 0.12 | <0.01 | 0.46 | ꭓ^2^ = 683.14,  df = 14,  *p* < 0.0001 | 0.03 |
| 20-24 | 0.31 | 0.26 | 0.04 | 0.15 | 0.01 |  |  |  |
| 25-29 | 0.64 | 0.57 | 0.07 | 0.12 | 0.01 |  |  |  |
| 30-34 | 1.17 | 1.10 | 0.07 | 0.06 | <0.01 |  |  |  |
| 35-39 | 1.75 | 1.75 | <0.01 | <0.01 | <0.01 |  |  |  |
| 40-44 | 2.76 | 2.78 | -0.03 | -0.01 | <0.01 |  |  |  |
| 45-49 | 4.58 | 4.41 | 0.17 | 0.04 | 0.01 |  |  |  |
| 50-54 | 7.77 | 6.82 | 0.95 | 0.13 | 0.12 |  |  |  |
| 55-59 | 11.23 | 10.58 | 0.65 | 0.06 | 0.04 |  |  |  |
| 60-64 | 13.91 | 14.19 | -0.29 | -0.02 | 0.01 |  |  |  |
| 65-69 | 16.54 | 16.34 | 0.19 | 0.01 | <0.01 |  |  |  |
| 70-74 | 13.84 | 15.27 | -1.43 | -0.10 | 0.14 |  |  |  |
| 75-79 | 10.47 | 11.40 | -0.94 | -0.09 | 0.08 |  |  |  |
| 80-84 | 7.46 | 7.48 | -0.02 | <0.01 | <0.01 |  |  |  |
| 85+ | 7.46 | 6.93 | 0.53 | 0.07 | 0.04 |  |  |  |
| **Sex** | | | | | | |  |  |
| Male | 52.25 | 51.59 | 0.66 | 0.01 | 0.01 | 0.02 | ꭓ^2^=25.99,  df = 1,  *p* < 0.0001 | 0.01 |
| Female | 47.75 | 48.41 | -0.66 | -0.01 | 0.01 |  |  |  |
| **Cancer group** | | | | | | |  |  |
| Breast | 13.10 | 13.23 | -0.13 | -0.01 | <0.01 | 0.11 | ꭓ^2^ = 278.96,  df = 3, *p* < 0.0001 | 0.02 |
| Colorectal | 22.33 | 21.34 | 0.99 | 0.05 | 0.04 |  |  |  |
| Genitourinary | 49.85 | 51.19 | -1.34 | -0.03 | 0.04 |  |  |  |
| Lung | 10.08 | 9.56 | 0.52 | 0.05 | 0.03 |  |  |  |
| Melanoma | 4.64 | 4.69 | -0.04 | -0.01 | <0.01 |  |  |  |

eTable 15. U.S. cancer population differences by age, sex, and cancer groups between year 2016 and 2020.

| Groups | 2016 | 2020 | Difference | Natural logarithm | PSI | Total PSI | Chi-square | Effect size |
| --- | --- | --- | --- | --- | --- | --- | --- | --- |
| **Age** | | | | | | |  |  |
| 15-19 | 0.12 | 0.11 | 0.01 | 0.05 | <0.01 | 0.64 | ꭓ^2^ = 900.81,  df = 14,  *p* < 0.0001 | 0.04 |
| 20-24 | 0.31 | 0.27 | 0.04 | 0.13 | <0.01 |  |  |  |
| 25-29 | 0.64 | 0.57 | 0.07 | 0.12 | 0.01 |  |  |  |
| 30-34 | 1.17 | 1.16 | 0.01 | 0.01 | <0.01 |  |  |  |
| 35-39 | 1.75 | 1.81 | -0.06 | -0.04 | <0.01 |  |  |  |
| 40-44 | 2.76 | 2.83 | -0.07 | -0.03 | <0.01 |  |  |  |
| 45-49 | 4.58 | 4.44 | 0.14 | 0.03 | <0.01 |  |  |  |
| 50-54 | 7.77 | 6.75 | 1.03 | 0.14 | 0.15 |  |  |  |
| 55-59 | 11.23 | 10.23 | 1.00 | 0.09 | 0.09 |  |  |  |
| 60-64 | 13.91 | 14.07 | -0.17 | -0.01 | <0.01 |  |  |  |
| 65-69 | 16.54 | 16.30 | 0.23 | 0.01 | <0.01 |  |  |  |
| 70-74 | 13.84 | 15.48 | -1.64 | -0.11 | 0.18 |  |  |  |
| 75-79 | 10.47 | 11.66 | -1.19 | -0.11 | 0.13 |  |  |  |
| 80-84 | 7.46 | 7.50 | -0.04 | -0.01 | <0.01 |  |  |  |
| 85+ | 7.46 | 6.80 | 0.66 | 0.09 | 0.06 |  |  |  |
| **Sex** | | | | | | |  |  |
| Male | 52.25 | 51.77 | 0.48 | 0.01 | <0.01 | 0.01 | ꭓ^2^ = 13.12,  df = 1,  *p* = 0.0003 | <0.01 |
| Female | 47.75 | 48.23 | -0.48 | -0.01 | <0.01 |  |  |  |
| **Cancer group** | | | | | | |  |  |
| Breast | 13.10 | 13.33 | -0.23 | -0.02 | <0.01 | 0.21 | ꭓ^2^ =511.07,  df = 3,  p < 0.0001 | 0.02 |
| Colorectal | 22.33 | 21.09 | 1.24 | 0.06 | 0.07 |  |  |  |
| Genitourinary | 49.85 | 51.80 | -1.94 | -0.04 | 0.07 |  |  |  |
| Lung | 10.08 | 9.36 | 0.72 | 0.07 | 0.05 |  |  |  |
| Melanoma | 4.64 | 4.43 | 0.21 | 0.05 | 0.01 |  |  |  |

eTable 16. U.S. cancer population differences by age, sex, and cancer groups between year 2017 and 2018.

| Groups | 2017 | 2018 | Difference | Natural logarithm | PSI | Total PSI | Chi-square | Effect size |
| --- | --- | --- | --- | --- | --- | --- | --- | --- |
| **Age** | | | | | | |  |  |
| 15-19 | 0.10 | 0.10 | -0.01 | -0.05 | <0.01 | 0.07 | ꭓ^2^ = 100.55,  df = 14,  *p* < 0.0001 | 0.01 |
| 20-24 | 0.28 | 0.27 | 0.01 | 0.02 | <0.01 |  |  |  |
| 25-29 | 0.62 | 0.58 | 0.04 | 0.07 | <0.01 |  |  |  |
| 30-34 | 1.10 | 1.09 | 0.01 | 0.01 | <0.01 |  |  |  |
| 35-39 | 1.73 | 1.75 | -0.02 | -0.01 | <0.01 |  |  |  |
| 40-44 | 2.72 | 2.75 | -0.02 | -0.01 | <0.01 |  |  |  |
| 45-49 | 4.59 | 4.49 | 0.10 | 0.02 | <0.01 |  |  |  |
| 50-54 | 7.38 | 7.09 | 0.29 | 0.04 | 0.01 |  |  |  |
| 55-59 | 11.00 | 10.66 | 0.34 | 0.03 | 0.01 |  |  |  |
| 60-64 | 14.20 | 14.25 | -0.05 | <0.01 | <0.01 |  |  |  |
| 65-69 | 16.45 | 16.22 | 0.23 | 0.01 | <0.01 |  |  |  |
| 70-74 | 14.59 | 14.96 | -0.37 | -0.02 | 0.01 |  |  |  |
| 75-79 | 10.60 | 11.14 | -0.54 | -0.05 | 0.03 |  |  |  |
| 80-84 | 7.47 | 7.51 | -0.04 | -0.01 | <0.01 |  |  |  |
| 85+ | 7.16 | 7.14 | 0.01 | <0.01 | <0.01 |  |  |  |
| **Sex** | | | | | | |  |  |
| Male | 51.93 | 52.01 | -0.08 | <0.01 | <0.01 | <0.01 | ꭓ^2^ = 0.35,  df = 1,  *p* = 0.553 | NA |
| Female | 48.07 | 47.99 | 0.08 | <0.01 | <0.01 |  |  |  |
| **Cancer group** | | | | | | |  |  |
| Breast | 13.12 | 13.27 | -0.15 | -0.01 | <0.01 | 0.01 | ꭓ^2^ = 18.49,  df = 3,  *p* = 0.0010 | <0.01 |
| Colorectal | 21.52 | 21.57 | -0.05 | <0.01 | <0.01 |  |  |  |
| Genitourinary | 50.80 | 50.84 | -0.04 | <0.01 | <0.01 |  |  |  |
| Lung | 9.91 | 9.69 | 0.23 | 0.02 | 0.01 |  |  |  |
| Melanoma | 4.64 | 4.63 | 0.01 | <0.01 | <0.01 |  |  |  |

eTable 17. U.S. cancer population differences by age, sex, and cancer groups between year 2017 and 2019.

| Groups | 2017 | 2019 | Difference | Natural logarithm | PSI | Total PSI | Chi-square | Effect size |
| --- | --- | --- | --- | --- | --- | --- | --- | --- |
| **Age** | | | | | | |  |  |
| 15-19 | 0.10 | 0.10 | -0.01 | -0.05 | <0.01 | 0.07 | ꭓ^2^ = 259.51,  df = 14, p < 0.0001 | 0.02 |
| 20-24 | 0.28 | 0.27 | 0.01 | 0.02 | <0.01 |  |  |  |
| 25-29 | 0.62 | 0.58 | 0.04 | 0.07 | <0.01 |  |  |  |
| 30-34 | 1.10 | 1.09 | 0.01 | 0.01 | <0.01 |  |  |  |
| 35-39 | 1.73 | 1.75 | -0.02 | -0.01 | <0.01 |  |  |  |
| 40-44 | 2.72 | 2.75 | -0.02 | -0.01 | <0.01 |  |  |  |
| 45-49 | 4.59 | 4.49 | 0.10 | 0.02 | <0.01 |  |  |  |
| 50-54 | 7.38 | 7.09 | 0.29 | 0.04 | 0.01 |  |  |  |
| 55-59 | 11.00 | 10.66 | 0.34 | 0.03 | 0.01 |  |  |  |
| 60-64 | 14.20 | 14.25 | -0.05 | <0.01 | <0.01 |  |  |  |
| 65-69 | 16.45 | 16.22 | 0.23 | 0.01 | <0.01 |  |  |  |
| 70-74 | 14.59 | 14.96 | -0.37 | -0.02 | 0.01 |  |  |  |
| 75-79 | 10.60 | 11.14 | -0.54 | -0.05 | 0.03 |  |  |  |
| 80-84 | 7.47 | 7.51 | -0.04 | -0.01 | <0.01 |  |  |  |
| 85+ | 7.16 | 7.14 | 0.01 | <0.01 | <0.01 |  |  |  |
| **Sex** | | | | | | |  |  |
| Male | 51.93 | 52.01 | -0.08 | <0.01 | <0.01 | <0.01 | ꭓ^2^ = 6.93, df = 1, p = 0.0085 | <0.01 |
| Female | 48.07 | 47.99 | 0.08 | <0.01 | <0.01 |  |  |  |
| **Cancer group** | | | | | | |  |  |
| Breast | 13.12 | 13.27 | -0.15 | -0.01 | <0.01 | 0.01 | ꭓ^2^ = 49.55,  df = 3,  *p* < 0.0001 | 0.01 |
| Colorectal | 21.52 | 21.57 | -0.05 | <0.01 | <0.01 |  |  |  |
| Genitourinary | 50.80 | 50.84 | -0.04 | <0.01 | <0.01 |  |  |  |
| Lung | 9.91 | 9.69 | 0.23 | 0.02 | 0.01 |  |  |  |
| Melanoma | 4.64 | 4.63 | 0.01 | <0.01 | <0.01 |  |  |  |

eTable 18. U.S. cancer population differences by age, sex, and cancer groups between year 2017 and 2020.

| Groups | 2017 | 2020 | Difference | Natural logarithm | PSI | Total PSI | Chi-square | Effect size |
| --- | --- | --- | --- | --- | --- | --- | --- | --- |
| **Age** | | | | | | |  |  |
| 15-19 | 0.10 | 0.10 | -0.01 | -0.05 | <0.01 | 0.07 | ꭓ^2^ = 439.42,  df = 14,  *p* < 0.0001 | 0.03 |
| 20-24 | 0.28 | 0.27 | 0.01 | 0.02 | <0.01 |  |  |  |
| 25-29 | 0.62 | 0.58 | 0.04 | 0.07 | <0.01 |  |  |  |
| 30-34 | 1.10 | 1.09 | 0.01 | 0.01 | <0.01 |  |  |  |
| 35-39 | 1.73 | 1.75 | -0.02 | -0.01 | <0.01 |  |  |  |
| 40-44 | 2.72 | 2.75 | -0.02 | -0.01 | <0.01 |  |  |  |
| 45-49 | 4.59 | 4.49 | 0.10 | 0.02 | <0.01 |  |  |  |
| 50-54 | 7.38 | 7.09 | 0.29 | 0.04 | 0.01 |  |  |  |
| 55-59 | 11.00 | 10.66 | 0.34 | 0.03 | 0.01 |  |  |  |
| 60-64 | 14.20 | 14.25 | -0.05 | <0.01 | <0.01 |  |  |  |
| 65-69 | 16.45 | 16.22 | 0.23 | 0.01 | <0.01 |  |  |  |
| 70-74 | 14.59 | 14.96 | -0.37 | -0.02 | 0.01 |  |  |  |
| 75-79 | 10.60 | 11.14 | -0.54 | -0.05 | 0.03 |  |  |  |
| 80-84 | 7.47 | 7.51 | -0.04 | -0.01 | <0.01 |  |  |  |
| 85+ | 7.16 | 7.14 | 0.01 | <0.01 | <0.01 |  |  |  |
| **Sex** | | | | | | |  |  |
| Male | 51.93 | 52.01 | -0.08 | <0.01 | <0.01 | <0.01 | ꭓ^2^ = 1.47,  df = 1,  *p* = 0.2258 | NA |
| Female | 48.07 | 47.99 | 0.08 | <0.01 | <0.01 |  |  |  |
| **Cancer group** | | | | | | |  |  |
| Breast | 13.12 | 13.27 | -0.15 | -0.01 | <0.01 | 0.01 | ꭓ^2^ = 180.76,  df = 3,  *p* < 0.0001 | 0.01 |
| Colorectal | 21.52 | 21.57 | -0.05 | <0.01 | <0.01 |  |  |  |
| Genitourinary | 50.80 | 50.84 | -0.04 | <0.01 | <0.01 |  |  |  |
| Lung | 9.91 | 9.69 | 0.23 | 0.02 | 0.01 |  |  |  |
| Melanoma | 4.64 | 4.63 | 0.01 | <0.01 | <0.01 |  |  |  |

eTable 19. U.S. cancer population differences by age, sex, and cancer groups between year 2018 and 2019.

| Groups | 2018 | 2019 | Difference | Natural logarithm | PSI | Total PSI | Chi-square | Effect size |
| --- | --- | --- | --- | --- | --- | --- | --- | --- |
| **Age** | | | | | | |  |  |
| 15-19 | 0.10 | 0.10 | <0.01 | -0.01 | <0.01 | 0.03 | ꭓ^2^ = 51.20,  df = 14,  *p* < 0.0001 | 0.01 |
| 20-24 | 0.27 | 0.26 | 0.01 | 0.02 | <0.01 |  |  |  |
| 25-29 | 0.58 | 0.57 | 0.01 | 0.02 | <0.01 |  |  |  |
| 30-34 | 1.09 | 1.10 | -0.01 | -0.01 | <0.01 |  |  |  |
| 35-39 | 1.75 | 1.75 | <0.01 | 0.00 | <0.01 |  |  |  |
| 40-44 | 2.75 | 2.78 | -0.04 | -0.01 | <0.01 |  |  |  |
| 45-49 | 4.49 | 4.41 | 0.08 | 0.02 | <0.01 |  |  |  |
| 50-54 | 7.09 | 6.82 | 0.27 | 0.04 | 0.01 |  |  |  |
| 55-59 | 10.66 | 10.58 | 0.08 | 0.01 | <0.01 |  |  |  |
| 60-64 | 14.25 | 14.19 | 0.06 | <0.01 | <0.01 |  |  |  |
| 65-69 | 16.22 | 16.34 | -0.13 | -0.01 | <0.01 |  |  |  |
| 70-74 | 14.96 | 15.27 | -0.31 | -0.02 | 0.01 |  |  |  |
| 75-79 | 11.14 | 11.40 | -0.26 | -0.02 | 0.01 |  |  |  |
| 80-84 | 7.51 | 7.48 | 0.03 | <0.01 | <0.01 |  |  |  |
| 85+ | 7.14 | 6.93 | 0.21 | 0.03 | 0.01 |  |  |  |
| **Sex** | | | | | | |  |  |
| Male | 52.01 | 51.59 | 0.42 | 0.01 | <0.01 | 0.01 | ꭓ^2^ = 10.50,  df = 1,  *p* = 0.0012 | <0.01 |
| Female | 47.99 | 48.41 | -0.42 | -0.01 | <0.01 |  |  |  |
| **Cancer group** | | | | | | |  |  |
| Breast | 13.27 | 13.23 | 0.04 | <0.01 | <0.01 | 0.01 | ꭓ^2^ = 19.60,  df = 3,  *p* = 0.0006 | <0.01 |
| Colorectal | 21.57 | 21.34 | 0.24 | 0.01 | <0.01 |  |  |  |
| Genitourinary | 50.84 | 51.19 | -0.35 | -0.01 | <0.01 |  |  |  |
| Lung | 9.69 | 9.56 | 0.13 | 0.01 | <0.01 |  |  |  |
| Melanoma | 4.63 | 4.69 | -0.05 | -0.01 | <0.01 |  |  |  |

eTable 20. U.S. cancer population differences by age, sex, and cancer groups between year 2018 and 2020.

| Groups | 2018 | 2020 | Difference | Natural logarithm | PSI | Total PSI | Chi-square | Effect size |
| --- | --- | --- | --- | --- | --- | --- | --- | --- |
| **Age** | | | | | | |  |  |
| 15-19 | 0.10 | 0.11 | -0.01 | -0.08 | <0.01 | 0.10 | ꭓ^2^ = 150.49, df = 14, *p* < 0.0001 | 0.02 |
| 20-24 | 0.27 | 0.27 | <0.01 | -0.01 | <0.01 |  |  |  |
| 25-29 | 0.58 | 0.57 | 0.01 | 0.02 | <0.01 |  |  |  |
| 30-34 | 1.09 | 1.16 | -0.06 | -0.06 | <0.01 |  |  |  |
| 35-39 | 1.75 | 1.81 | -0.06 | -0.04 | <0.01 |  |  |  |
| 40-44 | 2.75 | 2.83 | -0.09 | -0.03 | <0.01 |  |  |  |
| 45-49 | 4.49 | 4.44 | 0.04 | 0.01 | <0.01 |  |  |  |
| 50-54 | 7.09 | 6.75 | 0.35 | 0.05 | 0.02 |  |  |  |
| 55-59 | 10.66 | 10.23 | 0.42 | 0.04 | 0.02 |  |  |  |
| 60-64 | 14.25 | 14.07 | 0.18 | 0.01 | <0.01 |  |  |  |
| 65-69 | 16.22 | 16.30 | -0.09 | -0.01 | <0.01 |  |  |  |
| 70-74 | 14.96 | 15.48 | -0.52 | -0.03 | 0.02 |  |  |  |
| 75-79 | 11.14 | 11.66 | -0.52 | -0.05 | 0.02 |  |  |  |
| 80-84 | 7.51 | 7.50 | <0.01 | <0.01 | <0.01 |  |  |  |
| 85+ | 7.14 | 6.80 | 0.34 | 0.05 | 0.02 |  |  |  |
| **Sex** | | | | | | |  |  |
| Male | 52.01 | 51.77 | 0.24 | <0.01 | <0.01 | <0.01 | ꭓ^2^ = 3.24,  df = 1,  *p* = 0.0720 | NA |
| Female | 47.99 | 48.23 | -0.24 | <0.01 | <0.01 |  |  |  |
| **Cancer group** | | | | | | |  |  |
| Breast | 13.27 | 13.33 | -0.06 | <0.01 | <0.01 | 0.05 | ꭓ^2^ = 121.03,  df = 3,  *p* < 0.0001 | 0.01 |
| Colorectal | 21.57 | 21.09 | 0.48 | 0.02 | 0.01 |  |  |  |
| Genitourinary | 50.84 | 51.80 | -0.96 | -0.02 | 0.02 |  |  |  |
| Lung | 9.69 | 9.36 | 0.33 | 0.03 | 0.01 |  |  |  |
| Melanoma | 4.63 | 4.43 | 0.20 | 0.04 | 0.01 |  |  |  |

eTable 21. U.S. cancer population differences by age, sex, and cancer groups between year 2019 and 2020.

| Groups | 2019 | 2020 | Difference | Natural logarithm | PSI | Total PSI | Chi-square | Effect size |
| --- | --- | --- | --- | --- | --- | --- | --- | --- |
| **Age** | | | | | | |  |  |
| 15-19 | 0.10 | 0.11 | -0.01 | -0.07 | <0.01 | 0.03 | ꭓ^2^= 45.91, df = 14, p<0.0001 | 0.01 |
| 20-24 | 0.26 | 0.27 | -0.01 | -0.03 | <0.01 |  |  |  |
| 25-29 | 0.57 | 0.57 | <0.01 | <0.01 | <0.01 |  |  |  |
| 30-34 | 1.10 | 1.16 | -0.06 | -0.05 | <0.01 |  |  |  |
| 35-39 | 1.75 | 1.81 | -0.07 | -0.04 | <0.01 |  |  |  |
| 40-44 | 2.78 | 2.83 | -0.05 | -0.02 | <0.01 |  |  |  |
| 45-49 | 4.41 | 4.44 | -0.04 | -0.01 | <0.01 |  |  |  |
| 50-54 | 6.82 | 6.75 | 0.07 | 0.01 | <0.01 |  |  |  |
| 55-59 | 10.58 | 10.23 | 0.35 | 0.03 | 0.01 |  |  |  |
| 60-64 | 14.19 | 14.07 | 0.12 | 0.01 | <0.01 |  |  |  |
| 65-69 | 16.34 | 16.30 | 0.04 | <0.01 | <0.01 |  |  |  |
| 70-74 | 15.27 | 15.48 | -0.21 | -0.01 | <0.01 |  |  |  |
| 75-79 | 11.40 | 11.66 | -0.25 | -0.02 | 0.01 |  |  |  |
| 80-84 | 7.48 | 7.50 | -0.02 | <0.01 | <0.01 |  |  |  |
| 85+ | 6.93 | 6.80 | 0.13 | 0.02 | <0.01 |  |  |  |
| **Sex** | | | | | | |  |  |
| Male | 51.59 | 51.77 | -0.18 | <0.01 | <0.01 | <0.01 | ꭓ^2^ = 1.86,  df = 1,  *p* = 0.1730 | NA |
| Female | 48.41 | 48.23 | 0.18 | <0.01 | <0.01 |  |  |  |
| **Cancer group** | | | | | | |  |  |
| Breast | 13.23 | 13.33 | -0.10 | -0.01 | <0.01 | 0.03 | ꭓ^2^ = 72.83,  df = 3,  *p* < 0.0001 | 0.01 |
| Colorectal | 21.34 | 21.09 | 0.25 | 0.01 | <0.01 |  |  |  |
| Genitourinary | 51.19 | 51.80 | -0.60 | -0.01 | 0.01 |  |  |  |
| Lung | 9.56 | 9.36 | 0.20 | 0.02 | <0.01 |  |  |  |
| Melanoma | 4.69 | 4.43 | 0.26 | 0.06 | 0.01 |  |  |  |
